# Association of Cannabis Use Disorder with Major Adverse Cardiac and Cerebrovascular Events in Older Non-Tobacco Users: A Population-Based Analysis

**DOI:** 10.3390/medsci12010013

**Published:** 2024-02-19

**Authors:** Avilash Mondal, Sriharsha Dadana, Poojan Parmar, Maneeth Mylavarapu, Qiming Dong, Samia Rauf Butt, Abeera Kali, Bhaswanth Bollu, Rupak Desai

**Affiliations:** 1 Department of Internal Medicine, Nazareth Hospital, Philadelphia, PA 19152, USA; avilashmandal98@gmail.com; 2 Department of Hospital Medicine, Cheyenne Regional Medical Center, Cheyenne, WY 82001, USA; 3 Department of Medicine, Dr. M. K. Shah Medical College and Research Center, Ahmedabad 382424, India; poojanparmarmd@gmail.com; 4 Department of Public Health, Adelphi University, Garden City, NY 11530, USA; dr.maneeth.mylavarapu@gmail.com; 5 Department of Internal Medicine, Greater Baltimore Medical Center, Towson, MD 21204, USA; qdong@gbmc.org; 6 Department of Medicine, University College of Medicine and Dentistry, Lahore 55150, Pakistan; samia.butt38@yahoo.com; 7 Department of Medicine, Allama Iqbal Medical College, Lahore 54550, Pakistan; abeerakalimd@gmail.com; 8 Department of Emergency Medicine, All India Institute of Medical Sciences, New Delhi 110029, India; bhaswanthbollu@gmail.com; 9 Independent Researcher, Atlanta, GA 30033, USA; drrupakdesai@gmail.com

**Keywords:** cannabis use disorder/marijuana, tobacco use disorder/smokers, cardiovascular disease risk, major adverse cardiac and cerebrovascular events, acute myocardial infarction, stroke

## Abstract

Background: Tobacco use disorder (TUD) adversely impacts older patients with established cardiovascular disease (CVD) risk. However, CVD risk in chronic habitual cannabis users without the confounding impact of TUD hasn’t been explored. We aimed to determine the risk of major adverse cardiac and cerebrovascular events (MACCE) in older non-tobacco smokers with established CVD risk with vs. without cannabis use disorder (CUD). Methods: We queried the 2019 National Inpatient Sample for hospitalized non-tobacco smokers with established traditional CVD risk factors aged ≥65 years. Relevant ICD-10 codes were used to identify patients with vs. without CUD. Using multivariable logistic regression, we evaluated the odds of MACCE in CUD cohorts compared to non-CUD cohorts. Results: Prevalence of CUD in the sample was 0.3% (28,535/10,708,815, median age 69), predominantly male, black, and non-electively admitted from urban teaching hospitals. Of the older patients with CVD risk with CUD, 13.9% reported MACCE. The CUD cohort reported higher odds of MACCE (OR 1.20, 95% CI 1.11–1.29, *p* < 0.001) compared to the non-CUD cohort. Comorbidities such as hypertension (OR 1.9) and hyperlipidemia (OR 1.3) predicted a higher risk of MACCE in the CUD cohort. The CUD cohort also had higher unadjusted rates of acute myocardial infarction (7.6% vs. 6%) and stroke (5.2% vs. 4.8%). Conclusions: Among older non tobacco smokers with known CVD risk, chronic cannabis use had a 20% higher likelihood of MACCE compared to those who did not use cannabis.

## 1. Introduction

Medical and/or recreational cannabis use has been increasing rapidly since its legalization in the U.S. It started first in California in 1996 for its medicinal use, and the first recreational use was approved in the state of Washington 16 years later. Cannabis use in adults aged >65 years has taken a rise from 2.4% in 2015 to 4.2% in 2018 [1]. According to the National Poll of Healthy Aging conducted in 2021, 12.1% of the participants (50–80 years old) reported cannabis use, and among those who reported cannabis use, 34.2% reported cannabis use more than 3 times per week [2]. Numerous studies have shown a positive relationship between cannabis usage and adverse cardiovascular events [3]. There is increased hospitalization for acute myocardial infarction (AMI), arrhythmia, and stroke in cannabis users as compared to non-users, as indicated in some studies [4,5]. Studies have established cannabis and concomitant tobacco use as risk factors for major adverse cardiac and cerebrovascular complications in hospitalized patients [5]. Furthermore, studies have found that regular cannabis use is associated with an increased risk of death due to adverse cardiovascular diagnoses, particularly in non-tobacco users [6]. However, since tobacco use disorder (TUD) is commonly associated with cannabis use and a major cardiovascular disease (CVD) risk, it is important to eliminate the confounding effect of TUD and examine the independent impact of cannabis use on CVD risk and MACCE.

As the use of recreational cannabis becomes more prevalent and accepted, and given these concerning findings, it is crucial to understand the impact of regular cannabis use as an independent risk factor for CVD, especially in the geriatric age group. Hence, we conducted a large-scale analysis using the National Inpatient Sample (NIS), which allows for a comprehensive examination of the relationship between cannabis use, CVD risk, and MACCE. This study can provide valuable insights into the potential risks or benefits associated with cannabis use in older adults, ultimately contributing to informed decision-making in clinical practice and public health policies.

## 2. Methods

We conducted a retrospective analysis using the National Inpatient Sample (NIS) dataset (2019), provided by the Agency for Healthcare Research and Quality-sponsored Healthcare Cost and Utilization Project. NIS, the largest all-payer inpatient database (8 million annual hospital stays) in the United States, provides deidentified discharge data from a 20% stratified sample of discharges from community hospitals (excluding rehabilitation/long-term acute care hospitals), representing >95% of the U.S. population. Since the data are deidentified and publicly accessible, approval by the Institutional Review Board was not required.

We identified total admissions [age ≥ 65 years] in older patients with established CVD risk (defined by traditional CVD risk factors: hypertension, diabetes, hyperlipidemia, and obesity) without known TUD. We employed the relevant ICD-10 codes F12.90 for cannabis use disorder and Z72.0 for tobacco use to extract data from the NIS databases and categorized them into two groups: those with cannabis use disorder (CUD) and those without CUD (non-CUD). 

Primary outcomes were odds of major adverse cardiovascular and cerebrovascular events (MACCE) in CUD compared to non-CUD users. Additionally, we aimed to identify predictors of MACCE as our secondary outcomes. Logistic regression analysis was used to compare two categorical variables, assessed the odds of experiencing MACCE among the older patients in both the CUD and non-CUD cohorts. This analysis allowed us to evaluate the impact of CUD on the occurrence of MACCE, while accounting for other variables that could potentially confound the relationship. Furthermore, we conducted a secondary analysis to identify predictors of MACCE. This analysis aimed to identify additional factors or variables that could be associated with an increased likelihood of MACCE in older patients with CVD risk.

All analyses were conducted using weighted data and complex survey modules in IBM SPSS Statistics version 25.0 (IBM Corp., Armonk, NY, USA). For categorical data, we utilized the Pearson chi-square test, and for continuous variables, the Mann–Whitney U test [non-normal distribution curve]. Multivariable logistic regression analysis was performed to adjust for a number of potential confounding variables, such as age, race, sex, median household income national quartile, region of hospital, location/teaching status of hospital, comorbid conditions such as chronic pulmonary disease, acquired immune deficiency syndrome, alcohol abuse, depression, prior myocardial infarction (MI), drug abuse, prior transient ischemic attack (TIA) or stroke without neurologic deficit, prior venous thromboembolism, cancer, chronic kidney disease (CKD), hypertension, hyperlipidemia, obesity, diabetes, peripheral vascular disease (PVD), and other thyroid disorders. The *p*-value cutoff for statistical significance was 0.05.

## 3. Results

Of 10,708,814 total admissions with established CVD risk without known tobacco use disorder in patients of the geriatric age group, 28,535 (0.3%) were cannabis users. Non-cannabis users were older than cannabis users (median age 77 [IQR 71–84] vs. 69 [IQR 67–72] years, respectively). Males (69.5% vs. 45.8%), blacks (20.1 vs. 10.8%), lower median household income national quartile (31.6% vs. 27.0%), non-elective admissions (86.0% vs. 81.8%), urban teaching hospital admissions (76.1% vs. 70.9%), admissions from western region (36.3% vs. 18.2%), alcohol use (14.9% vs. 1.8%), uncomplicated hypertension (59.3% vs. 44.6%), prior history of myocardial infarction (11.3% vs. 9.1%), drug use (33.7% vs. 0.9%), and chronic pulmonary disease (30.8% vs. 25.2%) were more common among cannabis users compared to non-cannabis users. Conversely, females (30.5% vs. 54.2%), Hispanics (5.6% vs. 7.4%), Asian Pacific Islanders (0.7% vs. 2.7%), admissions from lower median household income national quartile (19.7% vs. 22.1%), diabetes with chronic complications (22.4% vs. 26.9%), diabetes without chronic complications (11.4% vs. 14.1%), and hyperlipidemia (55.5% vs. 60.4%) were less prevalent in cannabis users compared to non-cannabis users. All-cause mortality (3.3% vs. 1.7%) and dysrhythmia (34.9% vs. 24.9%) were higher in the non-CUD cohort compared to the CUD cohort; however, the rates were unadjusted. Acute myocardial infarction (7.6% vs. 6.0%, *p* = 0.001), transfer to other facilities (28.9% vs. 19.0%), and home health care disposition (22.4%) were higher in the CUD compared to non-CUD cohort (*p* = 0.001). Overall, after adjusting for baseline characteristics and comorbid variables, older patients with CUD had higher odds of having a MACCE during hospitalization (OR 1.20; 95% CI 1.11–1.29, *p* < 0.001) (Table 1).

On multivariable logistic regression, hypertension (OR 1.92; 95% CI 1.46–2.52, *p* < 0.001) and hyperlipidemia (OR 1.36; 95% CI 1.15–1.60, *p* < 0.001) were positive predictors whereas chronic kidney disease (OR 0.70; 95% CI 0.55–0.88, *p* = 0.002) were negative predictors of MACCE with statistical significance (Table 2).

## 4. Discussion

Our analysis of 2019 National Inpatient Sample data revealed that CUD is an independent risk factor for MACCE in hospitalized patients without a history of tobacco use disorder (TUD). Older patients with CVD risk and CUD had a MACCE incidence of almost 20% higher than the non-CUD group.

Recreational cannabis use has been implicated with arrhythmias, stroke [4,5,7,8], and heart failure [5]. Auer et al. [9] and Mittleman et al. [10] reported cannabis and concomitant tobacco use as risk factors for subclinical atherosclerosis. In contrast, our study found higher rates of MACCE (AMI, dysrhythmia, cardiac arrest, and stroke) in cannabis users even after excluding cases with tobacco use disorder. This raises doubts about the presumption that cardiovascular effects related to cannabis could be influenced by tobacco use disorder. The main reason behind these presumptions is cannabis and tobacco co-use. Studies have shown that cannabis users have an increased risk of using cigarettes and other tobacco-related products [11]. However, cannabis is equally potent in causing cardiovascular effects in the absence of smoking. Although the active ingredients (tetrahydrocannabinol vs. nicotine) in cannabis and tobacco are different, both of these produce a copious amount of chemicals when smoked, and these chemicals are predominantly identical. Furthermore, due to a different mode of smoking when compared to tobacco, cannabis chemicals are usually retained for a longer time in the body [12].

Stroke from cannabis use can be attributed to the procoagulant effect of its metabolite, delta-9-tetrahydrocannabinol, on platelets [13]. Its effect on short-term cerebral vasoconstriction is also a possible mechanism of stroke, especially in older individuals with increased CVD risk secondary to preexisting atherosclerosis [14]. Cannabis arteritis has already been described, given its deleterious effects on peripheral arteries, as something similar to Buerger’s disease, which is associated with tobacco smoking [15]. The Multicentric Study of Atherosclerosis, involving 1485 adult participants of age >65, similar to our study population, concluded that supraventricular tachycardia, premature atrial contractions, and non-sustained ventricular tachycardias were more frequently reported in regular cannabis users compared to never users [16]. The cardiovascular effects of cannabis use are not just acute, i.e., cannabis arteritis, cannabis-induced vasospasms, and platelet aggregation, but cannabis use is also hypothesized to be involved in atherosclerosis progression due to the abundance of CB1 and CB2 receptors (cannabinoid receptors) in the pulmonary and cardiovascular systems [17]. 

Conversely, Reis et al. [18] reported that 84% of adults (n = 5113 adults) had a history of using cannabis. The cumulative lifetime and recent cannabis use did not show an association with the incidence of CVD, stroke or TIA, coronary heart disease, or cardiac mortality. However, a comprehensive review conducted by Ravi et al. [19], involving 13 studies, concluded varied findings regarding the potential link between cannabis use and cardiovascular risk elements and outcomes like myocardial infarction and stroke. This is understandable because parallel randomized controlled trials would question ethicality, so all we can do is generate hypotheses based on observational studies. Using a nationally sampled database with enough samples, our study is unique in this sense. Notably, Chen et al. [20] introduced a novel dimension by investigating the genetic liability of CUD, revealing an association with an elevated risk of stroke, atrial fibrillation, heart failure, and pulmonary embolism, but the connection with coronary artery disease, myocardial infarction, and deep venous thrombosis remained weak. However, in our population-based analysis, patients with adverse cardiovascular and cerebrovascular events were almost twice as likely to be hypertensive and 30% more likely to be suffering from hyperlipidemia. Additionally, CUD patients were more likely to be male and of black origin. However, this population-based cross-sectional study pooling data from 2008–2017 concluded that legalization of cannabis was not associated with use disorder among blacks [21]. The mechanism of the increased rate of stroke in our study among a CUD cohort (5.2% vs. 4.8%, *p* = 0.002) who do not smoke tobacco remains to be established; however, it could be secondary to higher risk of hypertensive emergencies in older patients and arrhythmias [22,23].

Although we have substantial evidence to infer that CUD could be an independent risk factor for MACCE in older patients, including those who don’t use tobacco, the interpretation of this NIS database analysis should be conducted cautiously. It is essential to consider other factors that might influence the relationship between CUD and adverse cardiovascular outcomes. For instance, individuals with CUD may engage in other lifestyle habits or behaviors that could contribute to cardiovascular risk, since cannabis use is associated with increased substance abuse [11]. Also, the interaction between cannabis use and other pre-existing health conditions should be considered. It is important to acknowledge that a few of the patients might have had multiple hospitalizations during the period analyzed. Additionally, patients with CUD who were not hospitalized might not be a part of our study. Further research, especially prospective cohort studies and randomized control trials (RCTs), is imperative to establish a causal association between CUD and MACCE and elucidate the mechanistic effects of cannabis on the cardiovascular system. Additionally, basic science research focusing on various metabolic pathways is essential to understand the complete pathophysiology and long-term effects. Careful use of cannabis, both for recreational and medicinal purposes, is essential, especially in vulnerable age groups like the older population, and the CVD risk must be weighed against the benefits for use of cannabis in this age group. Furthermore, sustained research and data acquisition efforts would help clinicians and patients to make informed choices.

## 5. Conclusions

In conclusion, our research has unveiled a significant association between cannabis use and MACCE in older patients with a pre-existing CVD risk. Even after accounting for the potential confounding effect of concomitant tobacco smoking by excluding these cases, we observed a 20% higher likelihood of experiencing MACCE among older individuals with CVD risk who engaged in chronic or habitual cannabis use. This finding underscores the potential impact of cannabis use on the cardiovascular health of older patients with pre-existing heart-related conditions, highlighting the importance of further investigations into the association between CUD and adverse cardiovascular outcomes in this specific population. With the growing overlapping use of recreational and medicinal cannabis in the U.S. and its addiction potential, clinicians can play a crucial role in reducing associated risks by offering tailored interventions and guidance to protect the cardiovascular health of older individuals.

## Figures and Tables

**Table 1:** Baseline characteristics, outcomes of hospitalizations with vs. without CUD, NIS 2019.

Baseline Characteristics		Total Admissions with Cannabis Use Disorder		*p* Value
Variable		NO (n = 10,680,280)	YES (n = 28,535)	
Age (years) at admission	Median [IQR]	77 [71–84]	69 [67–72]	
Sex	Male	45.8%	69.5%	<0.001
	Female	54.2%	30.5%	
Race	White	76.4%	70.2%	<0.001
	Black	10.8%	20.1%	
	Hispanic	7.4%	5.6%	
	Asian or Pacific Islander	2.7%	0.7%	
	Native American	0.4%	1.0%	
Median household income national quartile for patient ZIP Code	0–25th	27.0%	31.6%	<0.001
	26–50th	25.5%	24.5%	
	51–75th	25.4%	24.1%	
	76–100th	22.1%	19.7%	
Non-elective admission		81.8%	86.0%	<0.001
Elective admission		18.2%	14.0%	
Hypertension, complicated		39.9%	33.6%	<0.001
Hypertension, uncomplicated		44.6%	53.9%	<0.001
Hyperlipidemia		60.4%	55.5%	<0.001
Obesity		17.8%	16.4%	<0.001
Drug abuse		0.9%	33.7%	<0.001
Chronic pulmonary disease		25.2%	30.8%	<0.001
Other thyroid disorders		1.4%	1.2%	0.009
Alcohol abuse		1.8%	14.9%	<0.001
Diabetes without chronic complications		14.1%	11.4%	<0.001
Prior myocardial infarction		9.1%	11.3%	<0.001
Prior transient ischemic attack/stroke		10.8%	9.7%	<0.001
Diabetes with chronic complications		26.9%	22.4%	<0.001
Prior VTE		6.4%	5.7%	<0.001
Disposition of patient	Routine	42.6%	56.0%	<0.001
	Other transfers SNF ICF	28.9%	19.0%	
	Home healthcare	22.4%	18.8%	
Length of stay (days)	Median [IQR]	4 [2–6]	4 [2–6]	0.490
Total charges (USD)	Median [IQR]	41,751 [22,924–77,379]	48,235 [25,299–90,831]	<0.001
**Outcomes**				
ACM		3.3%	1.7%	<0.001
AMI		6.0%	7.6%	<0.001
Dysrhythmia		34.9%	25.9%	<0.001
Cardiac Arrest		1.1%	1.1%	0.570
Stroke		4.8%	5.2%	0.002
	**Adjusted OR**	**95% CI Lower limit**	**95% CI Upper limit**	***p* Value**
MACCE (ACM, AMI, CA, and stroke)	1.20	1.11	1.29	<0.001

*p* < 0.05 indicates statistical significance. Abbreviations: VTE: venous thromboembolism, SNF: skilled nursing facility, ICF: intermediate care facility, AMI: acute myocardial infarction, ACM: all-cause mortality, MACCE: major adverse cardiovascular and cerebrovascular event, CA: cardiac arrest.

**Table 2:** Multivariate predictors of MACCE with cannabis use disorder among older non-tobacco users.

Predictor		aOR	95% CI UL	95% CI LL	*p* Value
Sex	Male vs. Female	1.15	0.96	1.37	0.13
Race	Black vs. White	1.23	1.00	1.52	0.17
	Hispanic vs. White	1.27	0.90	1.78	
	Asian or Pacific Islander vs. White	1.30	0.52	3.27	
	Native American vs. White	0.68	0.25	1.82	
	Others vs. White	0.68	0.35	1.32	
Median household income national quartile for patient ZIP Code	0–25th vs. 76–100th	0.97	0.76	1.23	0.57
	26–50th vs. 76–100th	0.92	0.72	1.17	
	51–75th vs. 76–100th	0.86	0.68	1.09	
Location/teaching status of hospital	Urban Non-teaching vs. Rural	0.83	0.54	1.28	0.02
	Urban Teaching vs. Rural	1.13	0.76	1.68	
Region of hospital	Midwest vs. Northeast	1.10	0.82	1.47	0.84
	South vs. Northeast	1.05	0.80	1.39	
	West vs. Northeast	1.12	0.85	1.47	
Chronic pulmonary disease	Present vs. Absent	0.74	0.63	0.88	0.00
Acquired immune deficiency syndrome	Present vs. Absent	0.36	0.13	1.01	0.05
Alcohol abuse	Present vs. Absent	1.13	0.90	1.41	0.28
Depression	Present vs. Absent	0.83	0.68	1.01	0.07
Drug abuse	Present vs. Absent	1.11	0.94	1.31	0.20
Renal failure	Present vs. Absent	0.70	0.55	0.88	0.00
Cancer	Present vs. Absent	0.88	0.67	1.15	0.34
Obesity	Present vs. Absent	0.83	0.67	1.04	0.11
Hypertension (Uncomplicated)	Present vs. Absent	0.87	0.67	1.12	0.28
Hypertension (Complicated)	Present vs. Absent	1.92	1.46	2.52	<0.001
Diabetes with chronic complications	Present vs. Absent	1.09	0.91	1.32	0.36
Diabetes without chronic complications	Present vs. Absent	0.95	0.74	1.23	0.72
Peripheral vascular disease	Present vs. Absent	1.26	1.00	1.60	0.05
Other thyroid disorders	Present vs. Absent	1.51	0.81	2.81	0.20
Hyperlipidemia	Present vs. Absent	1.36	1.15	1.60	<0.001
Prior Venous Thromboembolism	Present vs. Absent	0.68	0.45	1.02	0.06
Prior myocardial infarction	Present vs. Absent	1.15	0.92	1.44	0.23
Prior transient ischemic attack/stroke	Present vs. Absent	0.83	0.63	1.09	0.18

*p* < 0.05 indicates statistical significance. Multivariable regression models were adjusted for baseline demographics, hospital-level characteristics, and relevant cardiac and extra cardiac comorbidities.

## Data Availability

All data generated or analyzed during this study are included in this published article. Further data is avail-able from the corresponding author on reasonable request.
